# Rare Lesions Produced by Bromide of Potassium

**Published:** 1887-03

**Authors:** 


### *RARE LESIONS PRODUCED BY BROMIDE OF POTASSIUM.*

At a recent meeting of the New York Pathological Society, (*N. Y. Med. Journ.*, November 13, 1886,) Dr. R. W. Amidon presented an epileptic young woman, who had been under his care four years, taking moderate doses of bromide of potassium—perhaps never more than four or five drachms a day. A little over a year ago she discontinued it, but recently he learned that she had been taking during the past eighteen months a mixture of the bromides amounting to six drachms a day. She then returned to him, and he found the ordinary acne on the face, which, although usually confined to the face and neck, perhaps occasionally reaching to the chest and shoulders, had in this patient, as in two others whom he had seen, invaded the legs, and assumed almost the pustular form. The lesion began in the ordinary way, but became indurated with rather a large base, and ran a chronic course, breaking down and forming what appeared to be a small ulcer. Vesicles, with contents which tended to become cloudy and purulent, formed in rings about the acne spot. After a time the centre of the spot took on a reparative process, and entirely healed, while the pathological changes at the periphery spread, and the vesicles became purulent, dried up, and formed a brownish scab. A section of one of these spots showed that there had not been a true ulcer, but that the skin had simply been denuded of the cuticle, leaving the papillæ intact. He had in no case seen the true skin indurated. The disease did not seem to depend particularly upon the amount of the bromides given. The only treatment which he had seen do good was the thorough application of the actual cautery.

---

A VERY interesting contribution to our knowledge of peripheral neuritis in tabetic patients is found in the *Revue de Medecine*, No. 7, July 10, 1886, by Pitres and Vaillard. The conclusions of their memoir may be summed up as follows:

1. The peripheral nerves of tabetic patients are often the seat of neuritis.

2. The neuritis of tabetic patients does not differ in any essential respect from other forms of the non-traumatic affection.

3. Their topographical distribution in the body is variable, for the neuritis may attack the sensitive and mixed nerves and the visceral.

4. In the majority of cases, but not always, the disease begins at the outer extremity of the nerve.

5. Their extent and gravity have no constant relation in respect to age, or the extension or depth of the medullary regions of the locomotor ataxia.

6. It is probable they do not play any part in the production of the specific symptoms of tabes; such as, the lightning-like pain, incöordination of movements, abolition of patellar reflex, disorders of the muscular sense, etc. These latter symptoms depend rather upon the condition of the posterior columns of the cord.

7. Certain inconstant symptoms, however, which are added to or complicate the symptomatology of tabes, appear to depend upon the peripheral neuritis; such, for example, as anæsthetic spots in the skin, localized trophic disease of the skin and its dependencies, certain localized motor paralyses, accompanied or not by muscular atrophy, isolated joint affections, visceral crises, etc.—*Neurological Review.*

